# CMR atrial angiography makes redo AF ablations faster and easier with less x-ray fluoroscopy

**DOI:** 10.1186/1532-429X-11-S1-O88

**Published:** 2009-01-28

**Authors:** Andrew S Flett, Don M Milliken, Syed S Ahsan, James McReady, Pier D Lambiase, James C Moon

**Affiliations:** The Heart Hospital, London, UK

**Keywords:** Catheter Ablation, Fluoroscopy Time, Radiofrequency Catheter Ablation, Electroanatomic Mapping, Total Procedure Time

## Objective

To determine if merging CMR atrial angiograms with the fluoroscopy and ECG mapping during the ablation procedure make redo AF ablations easier.

## Background

AF ablations have a relatively low success rate (52–74%) and are long, complex procedures. CT merge into electroanatomic mapping in the catheter lab has been shown to produce better outcomes than using electroanatomic mapping alone. However, CT is associated with substantial radiation exposure. It is unclear whether CMR integration offers similar benefits. We hypothesised that CMR-derived 3D atrial anatomical merge would result in faster, easier procedures with less use of ionising radiation.

## Methods

64 patients (39 male, mean age: 57 +/- 12 years) underwent repeat radiofrequency catheter ablation of atrial fibrillation. Twenty-two (34%, the MERGE group) had a CMR merge, while 42 (66%, NO MERGE) did not. All patients underwent their procedure using the Ensite NavX system (St Jude Medical). The CMR atrial angiogram was performed prior to the procedure (non-gated, 3D atrial angiogram, 0.1 mmol/Kg contrast, timed for atrial delineation), and was available (non-subtracted) for importing into the cardiac catheterisation suite.

## Results

**Figure 1:**
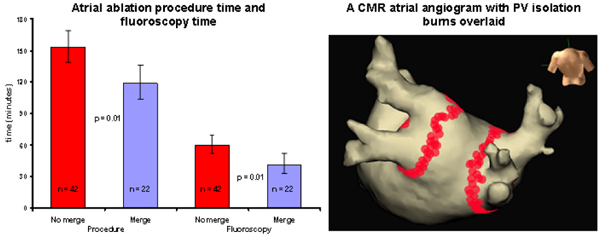
Figure 1

Compared to the NO MERGE group, the MERGE group demonstrated a substantial reduction in both total procedure time (34 minutes, 119 vs. 153 min; p = 0.01) and fluoroscopy time (19 minutes, 60 vs. 41 min; p = 0.01). In addition, there was a trend towards a reduction in left atrial mapping time (27 vs. 33 minutes; p = 0.25) and fewer radiofrequency lesions being required (204 vs. 250; p = 0.3). Figure 1

## Conclusion

CMR integration into electroanatomic mapping results in a reduction in procedure times and x-ray fluoroscopy in redo AF ablation compared to electroanatomic mapping alone. This is a potential benefit over CT image integration and electroanatomic mapping alone.

